# Interpreting single-step genomic evaluation as a neural network of three layers: pedigree, genotypes, and phenotypes

**DOI:** 10.1186/s12711-023-00838-7

**Published:** 2023-10-03

**Authors:** Tianjing Zhao, Hao Cheng

**Affiliations:** 1 Department of Animal Science, University of California Davis, Davis, CA 95616 USA; 2 Integrative Genetics and Genomics Graduate Group, University of California Davis, Davis, CA 95616 USA

## Abstract

The single-step approach has become the most widely-used methodology for genomic evaluations when only a subset of phenotyped individuals in the pedigree are genotyped, where the genotypes for non-genotyped individuals are imputed based on gene contents (i.e., genotypes) of genotyped individuals through their pedigree relationships. We proposed a new method named single-step neural network with mixed models (NNMM) to represent single-step genomic evaluations as a neural network of three sequential layers: pedigree, genotypes, and phenotypes. These three sequential layers of information create a unified network instead of two separate steps, allowing the unobserved gene contents of non-genotyped individuals to be sampled based on pedigree, observed genotypes of genotyped individuals, and phenotypes. In addition to imputation of genotypes using all three sources of information, including phenotypes, genotypes, and pedigree, single-step NNMM provides a more flexible framework to allow nonlinear relationships between genotypes and phenotypes, and for individuals to be genotyped with different single-nucleotide polymorphism (SNP) panels. The single-step NNMM has been implemented in the software package “JWAS’.

## Background

**Fig. 1:**
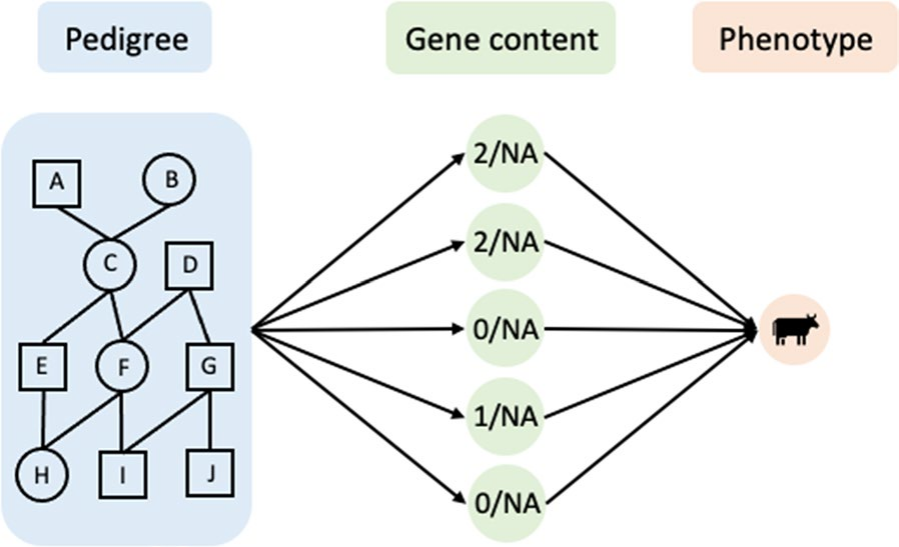
Framework of single-step NNMM with three fully-connected sequential layers of data: pedigree, genotypes, and phenotypes. Between the layer of pedigree and the layer of genotypes, the gene content of each marker is treated as a quantitative trait, and the pedigree is used to define the random effects covariance matrix. Each node in the middle layer represents the gene content of one marker. “NA” denotes missing values. For example, the nodes in the middle layer may be 2,2,0,1,0 for a genotyped individual or all missing (“NA”) for a non-genotyped individual. For non-genotyped individuals, all gene contents are missing and will be sampled conditional on pedigree, genotypes, and phenotypes in MCMC

The single-step approach [1–3] has been successfully adopted in genomic evaluations when only a subset of phenotyped individuals in the pedigree are genotyped. The single-step approach uses information from genotyped and non-genotyped relatives in two equivalent ways: (a) calculating an improved relationship matrix from pedigree and observed genotypes of genotyped individuals to model the covariances of breeding values for all relatives [1]; or equivalently, (b) imputing genotypes for non-genotyped individuals linearly based on gene contents (i.e., genotypes) of genotyped individuals and the pedigree, then propagating the uncertainty from the imputation by fitting additional random effects accounting for imputation errors in genomic evaluations [3] (see “Appendix”). In practice, the linear imputation in (b) can be obtained by modeling the gene content of each marker as a quantitative trait with a very high heritability and fitting the “expected” gene content as random effects based on covariances defined by the pedigree [4]. Thus, the latter interpretation (b) of the single-step approach, involves three sequential layers of information: pedigree, genotypes, and phenotypes. This leads to our new representation of the single-step approach as a neural network of three fully-connected sequential layers of information: pedigree (input layer), genotypes (middle layer), and phenotypes (output layer), as demonstrated in Fig. 1.

In previous work, we have proposed a method named “NNMM” (neural network with mixed models) for quantitative genetics, to extend mixed models (“MM”) to neural networks (“NN”) by adding intermediate layers of data (e.g., gene expression levels) between genotype and phenotype layers [5, 6]. Better prediction accuracies were observed when intermediate omics data were incorporated into genomic prediction using NNMM. In this paper, we show that NNMM can be adopted to incorporate pedigree, genotype, and phenotype information as a unified network named “single-step NNMM”, thus providing a new representation of the single-step approach, and yielding equivalent or higher prediction accuracies, due to the advantages described below.

Single-step NNMM has several advantages over the conventional single-step approach [1–3]. First, in the conventional single-step approach, gene contents of non-genotyped individuals are imputed based on the genotypes of genotyped individuals only through pedigree relationships. This can be considered as pre-analysis processing using Gengler’s method [4], and phenotypes are not included in this pre-analysis. We will show that, in single-step NNMM, such pre-analysis is not needed, and gene contents of non-genotyped individuals can be “imputed” based on pedigree, genotypes, and phenotypes in the Bayesian neural networks using Markov chain Monte Carlo (MCMC). Second, in single-step NNMM, the relationships between genotypes and phenotypes can be approximated by nonlinear activation functions of the neural network to introduce non-linearity between genotypes and phenotypes. Lastly, the conventional single-step approach requires individuals to be genotyped using the same single nucleotide polymorphism (SNP) panel (i.e., same markers for all genotyped individuals), while single-step NNMM can include individuals genotyped by different SNP panels (i.e., different markers for genotyped individuals) without pre-analysis.

In this paper, we present single-step NNMM for genomic evaluation, study its performance, and compare it to the conventional single-step approach [1–3]. Here, we focus on studying the effect of fitting pedigree, genotypes, and phenotypes jointly as three unified fully-connected sequential layers, in which gene contents of non-genotyped individuals are sampled conditional on all three layers of data. The same assumptions of linearity and of individuals being genotyped using the same SNP panel, as in the conventional single-step approach, were used in singe-step NNMM (i.e., a linear activation function, and individuals genotyped with the same SNP panel).

## Methods

In single-step NNMM, three sequential layers of information, i.e., pedigree, genotypes and phenotypes, form a unified neural network (instead of two separate steps) as demonstrated in Fig. 1. Mixed models were used to infer unknowns, including missing gene content of non-genotyped individuals and marker effects. In detail, at each iteration of the MCMC, unknowns will be sampled using Gibbs sampling from their full conditional posterior distributions at three levels: (1) from the input layer (pedigree) to the middle layer (gene contents): pedigree-based best linear unbiased prediction (PBLUP); (2) from the middle layer (gene contents) to the output layer (phenotypes): genomic BLUP (GBLUP) or Bayesian Alphabet; and (3) sampling missing values in the middle layer (genotypes for non-genotyped individuals) based on three layers of information, including pedigree, observed genotypes of genotyped individuals, and phenotypes.

### **From input layer (pedigree) to middle layer (gene contents): Pedigree-based BLUP**

Assuming there are *m* markers (i.e., *m* nodes in the middle layer), for the *j*th marker, the observed gene content (i.e., genotypes) of genotyped individuals can be modeled as:

1 $$\mathbf{z}_{g,j} = \mathbf{1}\mu _j + \mathbf{W}\mathbf{u}_j + \varvec{\varepsilon }_{j},$$

where $\mathbf{z}_{g,j}$ is a vector of observed gene contents (i.e., genotypes coded as 0/1/2) of marker *j* for genotyped individuals, and $\mu_j$ is its overall mean with a flat prior; $\mathbf{u}_j$ is the vector of gene content deviations (i.e., centered genotypes) for individuals in the pedigree with a prior $\mathbf{u}_j \sim MVN(\mathbf{0},\mathbf{A}\sigma ^2_{u_j})$, where the covariance matrix is the numerator relationship matrix of individuals in the pedigree ($\mathbf{A}$), scaled by variance component $\sigma ^2_{u_j}$; and $\mathbf{W}$ is the incidence matrix associating $\mathbf{u}_j$ with $\mathbf{z}_{g,j}$. The vector of random residuals, $\varvec{\varepsilon }_j$, is included to allow the use of mixed model equations, and to account for genotype or pedigree errors [4]. The prior of $\varvec{\varepsilon}_j$ is $\varvec{\varepsilon }_j \sim N(\mathbf{0}, \mathbf{I}\sigma ^2_{\epsilon _j})$. In principle, the heritability of gene content of each SNP $\left(\frac{\sigma ^2_{u_j}}{\sigma ^2_{u_j}+\sigma ^2_{\epsilon _j}}\right)$ should be 1 if the genotypes and pedigree information are perfectly correct and, thus, a small value of the estimated heritability indicates that there are errors in either genotypes or pedigree. Variance components are treated as unknowns in single-step NNMM, and scaled inverse chi-square distributions are assigned as prior distributions for variance components.

### **From middle layer (gene contents) to output layer (phenotypes): GBLUP or Bayesian Alphabet**

The phenotypes can be modeled as:

2 $$\mathbf{y}=\mathbf{1} \mu + \sum _{j=1}^{m} \mathbf{z}_j \alpha _j + \mathbf{e},$$

where $\mathbf{y}$ is the vector of phenotypes, $\mu$ is the overall mean with a flat prior, $\mathbf{z}_j$ is a vector of (observed and sampled) gene contents for the *j*th marker ($j=1,\ldots ,m$), and $\alpha _j$ is the corresponding marker effect. Priors from GBLUP [7–9] or the Bayesian Alphabet [10–18], such as BayesC$\pi$, can be used for sampling marker effects or breeding values. The vector $\mathbf{e}$ represents the residuals of phenotypes, with prior $\mathbf{e}\sim N(\mathbf{0}, \mathbf{I}\sigma ^2_e)$. The prior distribution of $\sigma ^2_e$ itself follows a scaled inverse chi-square distribution.

### **Sampling missing values in the middle layer (gene contents)**

Here we label the matrices related to non-genotyped and genotyped individuals with subscripts “n” and “g”, respectively. For the *j*th marker, the full conditional posterior distribution of the missing gene content is proportional to the product of its prior and the likelihood:

3 $$\begin{aligned}&f(\mathbf{z}_{n,j}| \mathbf{Z}_{n,-j}, \mathbf{Z}_{g}, \mathbf{y},\mathbf{A}, \mathbf{U}, ELSE) \\&\propto f(\mathbf{y} | \mathbf{Z}_{n}, \mathbf{Z}_{g},ELSE) f(\mathbf{z}_{n,j}, \mathbf{z}_{g,j}|\mathbf{u}_j, \mathbf{A},ELSE), \end{aligned}$$

where *ELSE* includes $\mu$, $\alpha _j$, $\sigma ^2_e$, $\mu _j$, $\sigma ^2_{u_j}$, and $\sigma ^2_{\epsilon _j}$ for $j=1,\dots ,m$, denoting the current values of all other unknowns except $\mathbf{Z}_{n}=[\mathbf{z}_{n,1},\ldots,\mathbf{z}_{n,m}]$ and $\mathbf{U}=[\mathbf{u}_{1},\ldots,\mathbf{u}_{m}]$. Detailed derivations are in “Appendix”.

When a nonlinear relationship is assumed between the middle layer (gene contents) and the output layer (phenotypes), Hamiltonian Monte Carlo (HMC) [19] may be employed for sampling missing genotypes. Note that if a linear relationship is assumed, missing genotypes can be sampled directly from a normal distribution at each iteration.

### **Data analysis**

Assuming linear relationships between genotypes (middle layer) and phenotypes (output layer), and that the same SNP panel is used for all genotyped individuals in the conventional single-step approach, we applied the same assumptions in the single-step NNMM (i.e., a linear activation function and individuals genotyped with the same SNP panel) to compare the prediction performance of these two methods. Thus, GBLUP was employed between the middle layer (gene contents) and the output layer (phenotypes) in the single-step NNMM (i.e., SS-NN-GBLUP), and its performance was compared to the conventional single-step GBLUP approach (i.e., SS-GBLUP).

The pig dataset from [20] was used, which includes 3534 genotyped individuals, and a pedigree of 6473 individuals including parents and grandparents of the genotyped animals. Estimates of heritability of gene content for each marker were close to 1 [21]. In our analysis, we used 10,000 randomly-selected SNPs as the genotype data. A random sample of 0.5%, i.e. 50, of these markers was selected as quantitative trait loci (QTL), and they were included in the genotypes. Phenotypes were simulated with a heritability of 0.7 and a phenotypic variance of 1. The 100 youngest individuals, whose genotypes were observed but phenotypes were unknown, were used for testing, while the remaining individuals (i.e., 3434 individuals) with known phenotypes were used for training.

To compare the single-step NNMM with the conventional single-step method in this study, different proportions of non-genotyped individuals in the training dataset were considered, including 30, 50, 70, and 90%, and there were 10 replicates for each scenario. For each replicate, individuals were randomly selected to be non-genotyped individuals. The prediction accuracy was calculated as the Pearson correlation between the true breeding values and the estimated breeding values for individuals in the testing dataset. In single-step NNMM, at least 2000 MCMC iterations were applied to ensure convergence.

In single-step NNMM, the heritability ($h^2$) of gene content in Eq. 1 can be considered as known to be 1 or unknown. When the heritability is considered known, a value close to 1 (i.e., $h^2=0.999$) is used to facilitate the use of mixed model equations. In single-step NNMM, two strategies were used to sample missing genotypes of non-genotyped individuals, i.e., missing genotypes were sampled conditionally on or unconditionally on phenotypes.

Unlike the conventional single-step approach, which requires individuals to be genotyped using the same SNP panel (i.e., identical markers for all genotyped individuals), the single-step NNMM can accommodate individuals genotyped with different SNP panels (i.e., varying markers for genotyped individuals). Thus, we also tested scenarios where SNP sets differed among individuals.

## Results

In single-step NNMM (SS-NN-GBLUP, i.e., single-step NNMM with GBLUP between middle and output layer), when the heritability ($h^2$) in Eq. 1 is considered unknown, the estimated heritability for each SNP was very close to 1.0, and similar results for SS-NN-GBLUP were observed regardless of whether the heritability ($h^2$) in Eq. 1 was assumed known (i.e., $h^2=1$) or unknown. Thus, only the results obtained with SS-NN-GBLUP with $h^2=1$ in Eq. 1 are presented. We compared the results of the conventional single-step method (SS-GBLUP, i.e., conventional single-step GBULP) and single-step NNMM (SS-NN-GBLUP) when missing genotypes of non-genotyped individuals were sampled conditionally on phenotypes, as described in Eq. 3. The results, presented in Table 1, demonstrate the prediction accuracy when various proportions of phenotyped individuals were genotyped. In general, the prediction accuracy of both methods decreased as the proportion of non-genotyped individuals increased. Overall, the SS-NN-GBLUP displayed a similar prediction accuracy to the SS-GBLUP approach, with no significant differences observed (pairwise t-test at a significance level of p < 0.01). The correlation between estimated marker effects from these two methods was high. Both the conventional single-step approach and single-step NNMM had significantly higher prediction accuracies compared to GBLUP using genotyped individuals only. The running time of SS-NN-GBLUP was less than 2 h using 20 central processing units (CPUs), while the conventional SS-GBLUP only took a few minutes (see “Discussion”). In addition, similar results were observed for SS-NN-GBLUP whether missing genotypes were sampled conditional on or unconditional on phenotypes.

**Table 1:** Comparison of prediction performances between conventional single-step (SS-GBLUP) and single-step NNMM (SS-NN-GBLUP)

Method	% non-genotyped individuals			
	30%	50%	70%	90%
SS-GBLUP	0.808 (0.005)	0.757 (0.007)	0.694 (0.013)	0.558 (0.015)
SS-NN-GBLUP	0.810 (0.006)	0.754 (0.007)	0.691 (0.013)	0.559 (0.014)

The average prediction accuracies from 10 replications with the standard deviation in brackets

We also tested scenarios where SNP sets differed among individuals, and the prediction accuracies aligned with our expectations. For example, when we randomly introduced 50% missing values in the genotype covariate matrix of the training dataset, the prediction accuracy was 0.767, with a standard deviation of 0.011 across 10 replications. This result is reasonable when compared to our previous findings. Note that when all individuals were genotyped, the prediction accuracy of GBLUP was 0.849.

## Discussion

In this paper, we propose a new method named single-step NNMM, which presents a novel framework for single-step methods by treating gene content (i.e., genotypes) as a middle layer of data between pedigree and phenotypes. Single-step NNMM represents single-step genomic evaluations as a neural network of three sequential layers: pedigree, genotypes, and phenotypes. Single-step NNMM is based on linear mixed models, i.e. PBLUP between the input layer (pedigree) and middle layer (gene content) and GBLUP/Bayesian Alphabet between the middle layer and the output layer (phenotype). This approach allows us to benefit from the implementation and optimization of well-studied linear mixed models for genomic prediction. Using the pedigree-based relationship matrix as an input of a neural network is not new. Gianola et al. [22] have shown that PBLUP is equivalent to a single (middle) layer neural network with a linear activation function, when the input is a pedigree-based relationship matrix. However, single-step NNMM extends conventional mixed models to a neural network with heterogeneous input data across multiple layers (more than two, i.e., pedigree, genotypes, phenotypes), whereas conventional mixed models or neural networks only consider two layers of data (input and output layers).

Compared to the conventional single-step method, the three sequential layers of information in single-step NNMM form a unified network, rather than two separate steps. Thus, the unobserved gene contents of non-genotyped individuals can be sampled based on information from all three layers: pedigree, observed genotypes of genotyped individuals, and phenotypes. Single-step NNMM offers a highly flexible framework for single-step methods, which allows nonlinear relationships between gene contents and phenotypes, as well as the genotyping of different individuals using distinct SNP panels (i.e., various patterns of missing genotypes). The single-step NNMM has been implemented in the software package “JWAS” [23, 24].

In our comparison, the same assumptions of linearity and identical SNP panels, as in conventional single-step approach, were used in singe-step NNMM. Overall, when some individuals were not genotyped, single-step NNMM had similar prediction accuracy as the conventional single-step approach, and the correlation between estimated marker effects from these two methods was high. Both conventional single-step approach and single-step NNMM had significantly higher prediction accuracies compared to GBLUP using genotyped individuals only.

As we have described, in addition to allowing non-linearity and individuals being genotyped with different SNP panels, a difference between single-step NNMM and the conventional single-step approach is in genotype imputation. Besides genotypes and pedigree, phenotypic information can also be used in the sampling of missing genotypes for non-genotyped individuals in single-step NNMM. However, similar prediction accuracies were observed regardless of whether missing genotypes were sampled conditional on or unconditional on phenotypes in SS-NN-GBLUP. For polygenic traits, this observation may be attributed to at least two reasons. First, a single SNP contributes only a small proportion of heritability and the correlation between the gene content of one SNP and phenotypes is generally low. As a result, incorporating phenotypic information into genotype imputation may introduce more noise than useful information. Second, phenotypes aid only in the imputation of causal variants, and variants in high linkage disequilibrium with causal variants. However, phenotypic information is employed in the imputation of all SNPs and can potentially introduce errors in marker imputation. However, when genotypes of relatives provide limited information (e.g., most individuals are not genotyped), the additional benefits in genotype imputation by including phenotypic information may not be negligible.

To enhance the applicability of our method to more realistic datasets, we implemented parallel computing using Message Passing Interface (MPI) [25], taking advantage of multiple computer processors’ capabilities. Ideally, with a sufficient number of computer processors, the computation time from the input layer (pedigree) to the middle layer (gene content) would be equal to the time required for one PBLUP, which should be relatively fast. The speed improvement from parallel computing is limited, however, by the hardware used. In our analysis of the pig dataset (i.e., 6473 individuals in the pedigree, 10,000 SNPs, and 3534 individuals with genotypes), running 2000 MCMC iterations on this dataset using 20 central processing units (CPUs) took less than 2 h for single-step NNMM, while the conventional single-step approach only took a few minutes. In future research, we plan to explore the use of graphics processing units (GPUs), which are commonly employed in neural networks, and more advanced parallel computing strategies (e.g., [26, 27]).

## Data Availability

Pig genotypes and pedigree used in the analysis are publicly available in [20]. The simulated phenotypes and all scripts are available at https://github.com/zhaotianjing/SSNNMM. The authors state that all data necessary for confirming the conclusions presented in the article are represented fully within the article.

## Appendix

### **Conventional single-step approach as linear imputations**

In a conventional single-step approach, a pre-analysis processing is used to impute the gene contents of non-genotyped individuals from the genotypes of genotyped individuals through pedigree relationships. In detail, the *centered* gene content of each marker is treated as a normally-distributed quantitative trait as $$\begin{bmatrix} \mathbf{z}_{n,j} \\ \mathbf{z}_{g,j} \end{bmatrix} \sim MVN(\mathbf{0},\mathbf{A}2p_ j q_j)$$, where $p_j$ is the allele frequency and $q_j=1-p_j$. Under Hardy–Weinberg equilibrium, the variance of marker is $2p_j q_j$. Thus, the distribution of $\mathbf{z}_{n,j}$ conditional on $\mathbf{z}_{g,j}$ is a multivariate normal distribution:

4 $$\begin{aligned} \mathbf{z}_{n,j}|\mathbf{z}_{g,j} \sim N(\mathbf{A}_{ng} \mathbf{A}_{gg}^{-1} \mathbf{z}_{g,j}, (\mathbf{A}_{nn}-\mathbf{A}_{ng} \mathbf{A}_{gg}^{-1} \mathbf{A}_{gn})2p_j q_j ). \end{aligned}$$

This can be written as:

5 $$\mathbf{z}_{n,j}=\hat{\mathbf{z}}_{n,j} + \mathbf{r}_{n,j},$$

where $\hat{\mathbf{z}}_{n,j}=\mathbf{A}_{ng} \mathbf{A}_{gg}^{-1} \mathbf{z}_{g,j}$ is the imputed genotypes for non-genotyped individuals, and $\mathbf{r}_{n,j}$ is the imputation uncertainty with $var(\mathbf{r}_{n,j})= (\mathbf{A}_{nn}-\mathbf{A}_{ng} \mathbf{A}_{gg}^{-1} \mathbf{A}_{gn})2p_j q_j$. Extending the above equation to multiple markers, we have $\hat{\mathbf{Z}}_{n}=\mathbf{A}_{ng} \mathbf{A}_{gg}^{-1} \mathbf{Z}_{g}$.

After the pre-analysis processing, both imputed and observed genotypes will be used for genomic evaluation. An additional random effect will be used to account for the uncertainty from the genotype imputation. In detail, the phenotypes are written as:

6 $$\begin{aligned} \begin{bmatrix} \mathbf{y}_{n} \\ \mathbf{y}_{g} \end{bmatrix}&= \mathbf{1} \mu + \begin{bmatrix} \sum _{j=1}^{m} \mathbf{z}_{n,j} \alpha _j \\ \sum _{j=1}^{m} \mathbf{z}_{g,j} \alpha _j \end{bmatrix}+ \mathbf{e}\\&= \mathbf{1} \mu + \begin{bmatrix} \sum _{j=1}^{m} (\hat{\mathbf{z}}_{n,j} \alpha _j + \mathbf{r}_{n,j} \alpha _j)\\ \mathbf{Z}_{g} \varvec{\alpha } \end{bmatrix} + \mathbf{e} \\&= \mathbf{1} \mu + \begin{bmatrix} \hat{\mathbf{Z}}_{n} \\ \mathbf{Z}_{g} \end{bmatrix} \varvec{\alpha } + \begin{bmatrix} \mathbf{r}^* \\ \mathbf{0} \end{bmatrix}+ \mathbf{e}, \end{aligned}$$

where $\mu$ is the overall mean, $\hat{\mathbf{Z}}_{n}$ is a matrix of imputed genotypes for non-genotyped individuals, $\mathbf{Z}_{g}$ is a matrix of observed genotypes for genotyped individuals, $\varvec{\alpha }$ is a vector of marker effects, and $\mathbf{e}$ is the vector of random residuals with $\mathbf{e} \sim MVN(\mathbf{0},\mathbf{I}\sigma ^2_{e})$. $\mathbf{r}^* = \sum _{j=1}^{m} \mathbf{r}_{n,j} \alpha _j$ is a random vector to account for the sum of weighted imputation uncertainty across all markers with variance:

7 $$\begin{aligned} var(\mathbf{r}^*)&= \sum _{j=1}^{m} var(\mathbf{r}_{n,j} \alpha _j)\\&= (\mathbf{A}_{nn}-\mathbf{A}_{ng} \mathbf{A}_{gg}^{-1} \mathbf{A}_{gn}) \sum _{j=1}^{m} 2p_j q_j \sigma ^2_{\alpha }\\&=(\mathbf{A}_{nn}-\mathbf{A}_{ng} \mathbf{A}_{gg}^{-1} \mathbf{A}_{gn})\sigma ^2_g, \end{aligned}$$

where $\sigma ^2_g=\sum _{j=1}^{m} 2p_j q_j \sigma ^2_{\alpha }$ is the genetic variance of phenotypes.

The conventional single-step GBLUP approach has been extended for Bayesian regression models to accommodate more flexible assumptions of the marker effects [3]. It has been shown that the single-step Bayesian regression model with a normal prior for marker effects is equivalent to the single-step GBLUP approach in terms of predicting genetic values.

### **Sampling missing genotypes**

Assuming a linear relationship between the middle layer (gene contents) and the output layer (phenotypes), the unobserved genotypes of non-genotyped individuals can be sampled from a normal distribution. Here we label the matrices related to non-genotyped and genotyped individuals with subscripts “n” and “g”, respectively.

For the *j*th marker ($j=1, \dots ,m$), let $n_n$ denote the number of non-genotyped individuals. The full conditional posterior distribution of unobserved genotypes for these non-genotyped individuals ($\mathbf{z}_{n,j}$) is as follows:

8 $$\begin{aligned} & f(\mathbf{z}_{n,j}| \mathbf{Z}_{n,-j}, \mathbf{Z}_{g}, \mathbf{y},\mathbf{A}, \mathbf{U},ELSE) \\ & \propto f(\mathbf{y} | \mathbf{Z}_{n}, \mathbf{Z}_{g},ELSE) f(\mathbf{Z}_{n}, \mathbf{Z}_{g},\mathbf{A},\mathbf{U},ELSE) \\ & \propto f(\mathbf{y}_n | \mathbf{Z}_{n}, ELSE ) f(\mathbf{y}_g| \mathbf{Z}_{g}, ELSE) \cdot \\ & \prod _{j=1}^{m} f(\mathbf{z}_{n,j}, \mathbf{z}_{g,j} | \mathbf{u}_j,ELSE) f(\mathbf{u}_j|\mathbf{A},ELSE) \\ & \propto f(\mathbf{y}_n | \mathbf{Z}_{n}, ELSE) f(\mathbf{z}_{n,j}, \mathbf{z}_{g,j} | \mathbf{u}_j,ELSE) f(\mathbf{u}_j|\mathbf{A},ELSE)\\ & \propto f(\mathbf{y}_n | \mathbf{Z}_{n}, ELSE) f(\mathbf{z}_{n,j} | \mathbf{u}_{n,j},ELSE) f(\mathbf{z}_{g,j} | \mathbf{u}_{g,j},ELSE) \cdot \\ & f(\mathbf{u}_j|\mathbf{A},ELSE)\\ & \propto f(\mathbf{y}_n | \mathbf{Z}_{n},ELSE) f(\mathbf{z}_{n,j} | \mathbf{u}_{n,j},ELSE)\\ & \propto (\sigma ^2_{e})^{-\frac{n_n}{2}} exp\left\{ \frac{ [\mathbf{c}_{n,j} - \mathbf{z}_{n,j} \alpha _{j}]^T [\mathbf{c}_{n,j} - \mathbf{z}_{n,j} \alpha _{j}] }{-2 \sigma ^2_{e}} \right \} . \\ & (\sigma ^2_{\epsilon _j})^{-\frac{n_n}{2}} exp \left\{ \frac{[\mathbf{z}_{n,j}-\mathbf{d}_{n,j}]^{T}[\mathbf{z}_{n,j} -\mathbf{d}_{n,j}] }{-2 \sigma ^2_{\epsilon _j}} \right \} \\ & \propto exp \left\{ \frac{\mathbf{z}_{n,j}^T\mathbf{z}_{n,j} \alpha ^2_j - 2 \mathbf{z}_{n,j}^T \mathbf{c}_{n,j} \alpha _j }{-2 \sigma ^2_e } \right\} exp \left \{ \frac{ \mathbf{z}_{n,j}^T\mathbf{z}_{n,j} - 2\mathbf{z}_{n,j}^T \mathbf{d}_{n,j} }{-2 \sigma ^2_{\epsilon _j}}\right \}\\ & = exp \left\{ \frac{\mathbf{z}_{n,j}^T\mathbf{z}_{n,j} \alpha ^2_j \sigma ^2_{\epsilon _j}- 2 \mathbf{z}_{n,j}^T \mathbf{c}_{n,j} \alpha _j \sigma ^2_{\epsilon _j}}{-2 \sigma ^2_e \sigma ^2_{\epsilon _j}}\right \} exp \left\{ \frac{ \mathbf{z}_{n,j}^T\mathbf{z}_{n,j} \sigma ^2_e - 2\mathbf{z}_{n,j}^T \mathbf{d}_{n,j} \sigma ^2_e}{-2 \sigma ^2_{\epsilon _j} \sigma ^2_e} \right\}\\ & = exp \left\{ \frac{\mathbf{z}_{n,j}^T\mathbf{z}_{n,j} (\alpha ^2_j \sigma ^2_{\epsilon _j} + \sigma ^2_e)- 2 \mathbf{z}_{n,j}^T (\mathbf{c}_{n,j} \alpha _j \sigma ^2_{\epsilon _j} + \mathbf{d}_{n,j} \sigma ^2_e)}{-2 \sigma ^2_e \sigma ^2_{\epsilon _j}}\right \}\\ & = exp \left\{ \frac{ \mathbf{z}_{n,j}^T\mathbf{z}_{n,j} -2 \mathbf{z}_{n,j}^T \left(\frac{\mathbf{c}_{n,j} \alpha _j \sigma ^2_{\epsilon _j}+ \mathbf{d}_{n,j} \sigma ^2_e}{\alpha ^2_j \sigma ^2_{\epsilon _j} + \sigma ^2_e}\right) }{-2 \frac{\sigma ^2_e \sigma ^2_{\epsilon _j}}{\alpha ^2_j \sigma^2_{\epsilon _j} + \sigma ^2_e}} \right\} \\ & = exp \left\{ \frac{ \mathbf{z}_{n,j}^T\mathbf{z}_{n,j} -2 \mathbf{z}_{n,j}^T \mathbf{f}_{n,j} }{-2 s_j^2}\right \} \\ & \sim MVN (\mathbf{f}_{n,j}, \mathbf{I} s^2_j), \end{aligned}$$

where $\mathbf{c}_{n,j} = \mathbf{y}_n - \mathbf{1}\mu - \sum _{j' \ne j} \mathbf{z}_{n,j'} \alpha _{j'}$, $\mathbf{d}_{n,j}=\mathbf{1}\mu _j+\mathbf{u}_{n,j}$, $\mathbf{f}_{n,j}= \frac{\mathbf{c}_{n,j} \alpha _j \sigma ^2_{\epsilon _j}+ \mathbf{d}_{n,j} \sigma ^2_e}{\alpha ^2_j \sigma ^2_{\epsilon _j} + \sigma ^2_e}$, $s^2_j=\frac{\sigma ^2_e \sigma ^2_{\epsilon _j}}{\alpha ^2_j \sigma ^2_{\epsilon _j} + \sigma ^2_e}$. The term *ELSE* represents unknowns, excluding $\mathbf{Z}_{n}=[\mathbf{z}_{n,1},\ldots,\mathbf{z}_{n,m}]$ and $\mathbf{U}=[\mathbf{u}_{1},\ldots,\mathbf{u}_{m}]$. It includes $\mu$, $\alpha _j$, $\sigma ^2_e$, $\mu _j$, $\sigma ^2_{u_j}$, and $\sigma ^2_{\epsilon _j}$ for $j=1,\dots ,m$.

From the full conditional posterior distribution of $\mathbf{z}_{n,j}$, it is evident that elements within $\mathbf{z}_{n,j}$ can be independently sampled from a univariate normal distribution. However, the full conditional posterior distribution of $\mathbf{z}_{n,j}$ is dependent on all other markers $\mathbf{z}_{n,j'}$ through the term $\mathbf{c}_{n,j} = \mathbf{y}_n - \mathbf{1}\mu - \sum _{j' \ne j} \mathbf{z}_{n,j'} \alpha _{j'}$. Consequently, missing genotypes for all markers cannot be sampled simultaneously.

### **Sampling missing genotypes (unconditional on phenotypes)**

Our results have indicated that phenotypic information provides limited improvement for genotype imputation. From a computational perspective, when sampling missing genotypes conditional on phenotypes, the full conditional posterior distribution of $\mathbf{z}_{n,j}$ depends on all other markers $\mathbf{z}_{n,j'}$ through the term $\mathbf{y}_n - \mathbf{1}\mu - \sum _{j' \ne j} \mathbf{z}_{n,j'} \alpha _{j'}$. This dependency makes the imputation of each marker dependent, which needs the use of approximations to enable parallel computing.

Therefore, below we present the sampling of missing gene content without conditioning on phenotypes. In this scenario, the single-step NNMM is identical to conventional single-step methods. The full conditional posterior distribution of $\mathbf{z}_{n,j}$ is as follows:

9 $$\begin{aligned}&f(\mathbf{z}_{n,j}| \mathbf{Z}_{n,-j}, \mathbf{Z}_{g},\mathbf{A}, \mathbf{U},ELSE) \\&\propto f(\mathbf{z}_{n,j} | \mathbf{u}_{n,j},ELSE)\\&\propto (\sigma ^2_{\epsilon _j})^{-\frac{n_n}{2}} exp \left\{ \frac{[\mathbf{z}_{n,j} -\mathbf{d}_{n,j}]^{T}[\mathbf{z}_{n,j}-\mathbf{d}_{n,j}] }{-2 \sigma ^2_{\epsilon _j}} \right \} \\&\sim MVN (\mathbf{d}_{n,j}, \mathbf{I} \sigma ^2_{\epsilon _j}), \end{aligned}$$

where $$\mathbf{d}_{n,j}=\mathbf{1}\mu _j+\mathbf{u}_{n,j}$$.

The full conditional posterior distribution of $\mathbf{z}_{n,j}$ is independent of $\mathbf{z}_{n,j'}$, allowing for simultaneous sampling of missing genotypes (i.e., $\mathbf{z}_{n,1}$, $\mathbf{z}_{n,2},\ldots, \mathbf{z}_{n,m}$).
